# Comprehensive analysis of a TPX2-related TRHDE-AS1/PKIA ceRNA network involving prognostic signatures in Hepatitis B virus-infected hepatocellular carcinoma

**DOI:** 10.3389/fcimb.2022.1025900

**Published:** 2022-09-20

**Authors:** Gaopeng Li, Zhuangqiang Wang, Dong Chen, Jun Yin, Zhiyuan Mo, Bianyin Sun, Tao Yang, Xinning Zhang, Zhensheng Zhai, Yaoxuan Li, Pinggui Chen, Yunyan Dai, Zhiming Wang, Jun Ma

**Affiliations:** General Surgery Department, Shanxi Bethune Hospital, Taiyuan, China

**Keywords:** hepatitis B virus, immune infiltration, ceRNA, prognosis, hepatocellular carcinoma

## Abstract

Hepatitis B virus (HBV) infection is a main carcinogenic factor of hepatocellular carcinoma (HCC). TPX2 microtubule nucleation factor is recently recommended as a novel prognostic biomarker in HBV-infected HCC tissues. This study aimed to explore a TPX2-related ceRNA regulatory network in HBV-infected HCC and the potential impact on HCC prognosis. We comprehensively identified 541 differential expressed lncRNAs (DElncRNAs), 37 DEmiRNAs and 439 DEmRNAs from HBV-related TCGA-HCC cohorts in TPX2^low^ and TPX2^high^ groups. Based on their RNA-RNA interaction and expression analysis, four DElncRNAs (TRHDE-AS1, DLX6-AS1, SNHG14, HOXA11-AS), four DEmiRNAs (miR-23b, miR-320a, miR-589, miR-126) and five DEmRNAs (PKIA, PCDHA2, SHCBP1, PRSS16, KIF18A) in HCC tumor vs normal groups were subjected to the hub regulatory networks analysis and further prognostic value analysis. Importantly, the TRHDE-AS1/miR-23b/PKIA ceRNA network was associated with HCC prognosis. Furthermore, cellular location analysis and base-base interaction analysis indicated that the cytoplasmic lncRNA TRHDE-AS1 was regarded as a ceRNA to sponging miR-23b and then regulating PKIA. Interestingly, correlation analysis suggested the expression correlation between TRHDE-AS1 and PKIA in HCC. Finally, we further performed the methylation and immune infiltration analysis to explore the functional process of PKIA in HCC. We proposed a ceRNA regulatory network may help elucidate the mechanism by which TPX2 contributes to the prognosis of HBV-related HCC.

## Introduction

Hepatocellular carcinoma (HCC) is a common primary liver cancer with high incidence and high mortality, accounting for 85% of liver cancer types (Sia et al., 2017). A 5-year survival rate of HCC patients is approximately 50%-60% (Llovet et al., 2003). Surgical partial hepatectomy is the first choice for treatment of resectable HCC cases (Lu and Dong, 2014). However, the 5-year recurrence rate of patients undergoing hepatectomy is still as high as 50-70% (Marasco et al., 2019). Hepatitis B virus (HBV), a member of the hepadnaviridae family, is a partially double-stranded DNA virus with a full length of 3200 bp (Tsukuda and Watashi, 2020). HBV infection is a major cause of serious liver-related diseases such as cirrhosis, liver failure and HCC (Seto et al., 2018). Elevated HBV DNA level is closely related to postoperative recurrence of HCC. A study indicated that antiviral therapy can reduce the activity of HBV and improve the overall survival rate of patients after radical resection of HCC (Wong et al., 2020). Hence, it is crucial to explore new biomarkers for the development and prognosis of HBV-related HCC.

Long non-coding RNAs (lncRNAs) participate in biological processes by controlling gene expression at the epigenetic, transcriptional and post-transcriptional levels, instead of translating into proteins (Mercer et al., 2009). Cytoplasmic lncRNAs can stabilize ribonucleoprotein complexes and act as competing endogenous RNAs (ceRNAs) for binding to miRNAs (Tay et al., 2014). Previous researches have reported the function of lncRNAs in different diseases, such as cancer, immune system diseases, and virus infection (Qi et al., 2015; Lu et al., 2020; Li et al., 2021). For example, lncRNA SNHG11/miR-184/AGO2 axis regulated cellular malignant processes, including proliferation, migration, and autophagy in HCC (Huang et al., 2020). Li et al. proposed that HULC/miR-200a-3p/ZEB1 network played an essential regulatory role in HCC epithelial-mesenchymal transition (EMT) process and tumor metastasis (Li et al., 2016). However, there is still no bioinformatics evidence on the regulation of ceRNA network in HBV-related HCC.

**Figure 1:**
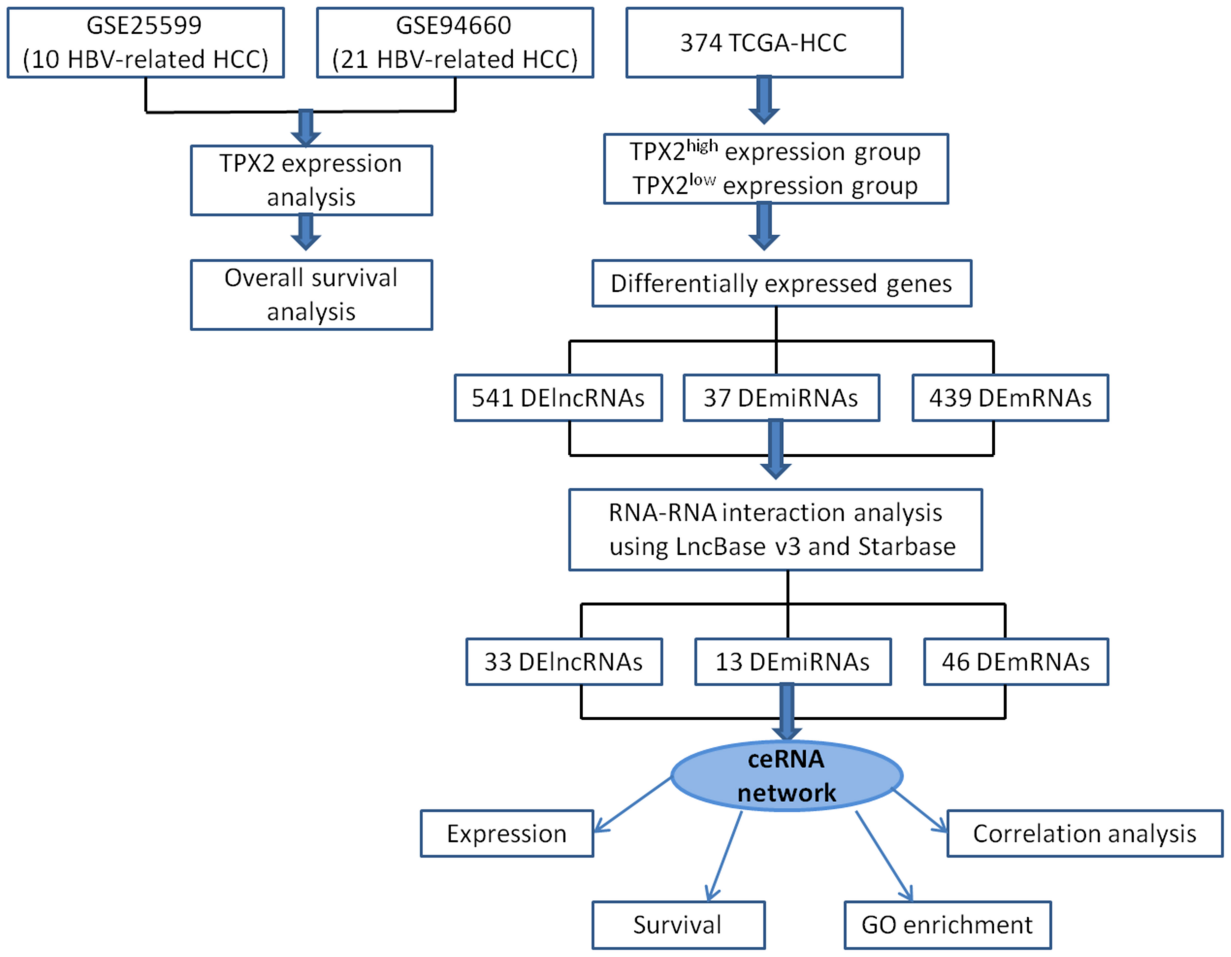
Flowchart of ceRNA network analysis in HBV-related HCC.

TPX2 microtubule nucleation factor is a microtubule-associated protein required for the formation of microtubules during cell mitosis (Neumayer et al., 2014). TPX2 recruits Aurora-A kinase to microtubules and drives the activation of Aurora-A kinase during mitosis (Asteriti et al., 2010). Accumulating analysis has shown that high expression of TPX2 is involved in worse prognosis of patients with HBV-related HCC (Zeng et al., 2020). In this research, we utilized the GEO RNA-seq data and the Cancer Genome Atlas (TCGA) HCC cohort to confirm the difference of TPX2 expression levels between HBV-related HCC tissues and normal samples, and screen differential expressed lncRNAs (DElncRNAs), DEmicroRNAs and DEmRNAs in TPX2^high^ vs TPX2^low^ expression groups (**Figure 1**). Next, by analyzing expression levels, overall survival, base-complementation of lncRNA-miRNA-mRNA and gene expression correlation, we comprehensively explored a potential TRHDE-AS1/PKIA ceRNA network by which TPX2 modulates in HBV-related HCC. Furthermore, we carried out the methylation and immune infiltration analysis to discuss the potential mechanism of PKIA regulation on HBV-HCC occurrence and prognosis. The findings about PTX2-related ceRNA patterns are helpful to discover the underlying mechanisms and to provide novel biomarkers for the prognosis of HBV-related HCC.

## Methods

### Data collection and gene expression analysis

The expression profiling by high throughput sequencing datasets (GSE25599 and GSE94660) were collected from the Gene Expression Omnibus (GEO, https://www.ncbi.nlm.nih.gov/gds/?term=). GSE25599 includes 10 match-paired HBV-related HCC and non-cancerous adjacent samples (Huang et al., 2011). GSE94660 includes 21 pairs of tumor and non-neoplastic liver tissues of HBV-HCC patients (Yoo et al., 2017). Raw RNA-seq data was obtained from TCGA-LIHC level 3 HTSeq-counts project (https://portal.gdc.cancer.gov/), which contains 50 cases with paracancerous tissues and 374 cases with HCC. The obtained counts data was converted into transcripts per million (TPM) format by using R software (version 3.6.3). TPX2 expression in pan-cancer was analyzed by using TCGA-All level 3 HTSeq-FPKM project and visualized by using ggplot2 (version 3.3.3). DElncRNAs, DEmiRNAs and DEmRNAs between TPX2^high^-HCC samples and TPX2^low^-HCC samples were identified with critical values of |LogFC| > 1.5, |LogFC| > 0.5 and |LogFC| > 2, respectively (all adjusted p value < 0.05). Volcano plots and heatmap were visualized by ggplot2. The immunohistochemical (IHC) results of TPX2 protein expression was obtained from the Human Protein Atlas (https://www.proteinatlas.org/). The antibody HPA005487 against TPX2 protein was used for IHC assay.

### Analysis of TPX2 and PKIA genetic alteration

cBioPortal online tool (https://www.cbioportal.org/) (Gao et al., 2013) was utilized to analyze TPX2 or PKIA mutations across Live Hepatocellular Carcinoma type. We selected “Mutations” and “Putative copy-number alterations from GISTIC” Genomic Profiles, and then entered gene “TPX2” and “PKIA”. OncoPrint module showed a schematic diagram including the percentage of genetic alternation. Plots module presented a scatter diagram containing the correlation of mRNA vs Copy number alteration (CNA).

### Analysis of overall survival and gene ontology (GO) enrichment

The survival R package (version 3.6.3) and survminer package (version 0.4.9) was downloaded to identify prognostic genes among DElncRNAs, DEmiRNAs and DEmRNAs. TCGA-HCC samples were divide into high and low expression groups. Survival package (version 3.2-10) was utilized for statistic analysis of overall survival. DEmRNAs were subjected to GO enrichment analysis using Database for annotation, visualization, and integrated discovery (DAVID, https://david.ncifcrf.gov/). The term counts and –log10pvalue for BP (Biological process), CC (Cellular component), and MF (Molecular function) were showed with box plot.

### Analysis of lncRNA-miRNA-mRNA network interaction

Using DIANA-LncBase v3 (https://diana.e-ce.uth.gr/lncbasev3) online tool, we explored the RNAs-RNAs interaction between DElncRNAs and DEmiRNAs. In addition, the DEmRNA targeted by DEmiRNAs was predicted using Starbase (https://starbase.sysu.edu.cn/). The primary ceRNA network was visualized by Cytoscape. The hub genes with score > 2 was identified by Cytohubba plug-in module. To explore the role of lncRNA TRHDE-AS1 in HCC, we obtained the sequence of TRHDE-AS1 in LNCipedia (https://lncipedia.org/) and confirmed the subcellular location in LncLocator (http://www.csbio.sjtu.edu.cn/bioinf/lncLocator/). RNAhybrid (https://bibiserv.cebitec.uni-bielefeld.de/rnahybrid) was utilized to calculate a minimal free energy (mfe) and predict the binding sites between RNAs and RNAs.

### Analysis of PKIA methylation

The expression levels DNA methyltransferases (DNMT1, DNMT3A, and DNMT3B) was investigated in PKIA^high^ and PKIA^low^-TCGA-HCC samples. UALCAN (http://ualcan.path.uab.edu/) online data analysis was performed to detect DNA methylation statue in HCC vs paracancerous samples. Furthermore, methylation sites in PKIA was analyzed using the MEXPRESS visualization tool (https://mexpress.be/).

### Immune infiltration analysis

The correlation between GLA expression level and infiltration level in multiple immune cells (B cell, myeloid dendritic cell, macrophage, monocyte, neutrophil and T cell) was analyzed on the Tumor Immune Estimation Resource (TIMER, (http://cistrome.org/TIMER/). In addition, we explored the clinical relevance of tumor immune subsets with PKIA expression levels in HCC using immure outcome module. The correlation between PKIA level with multiple immune markers was explored in Gene_Corr module. The degree of correlation was calculated with purity-adjusted partial spearman’s rho value.

## Results

### TPX2 is a differentially expressed gene (DEG) and prognostic biomarker in HBV-related HCC tissues

**Figure 2:**
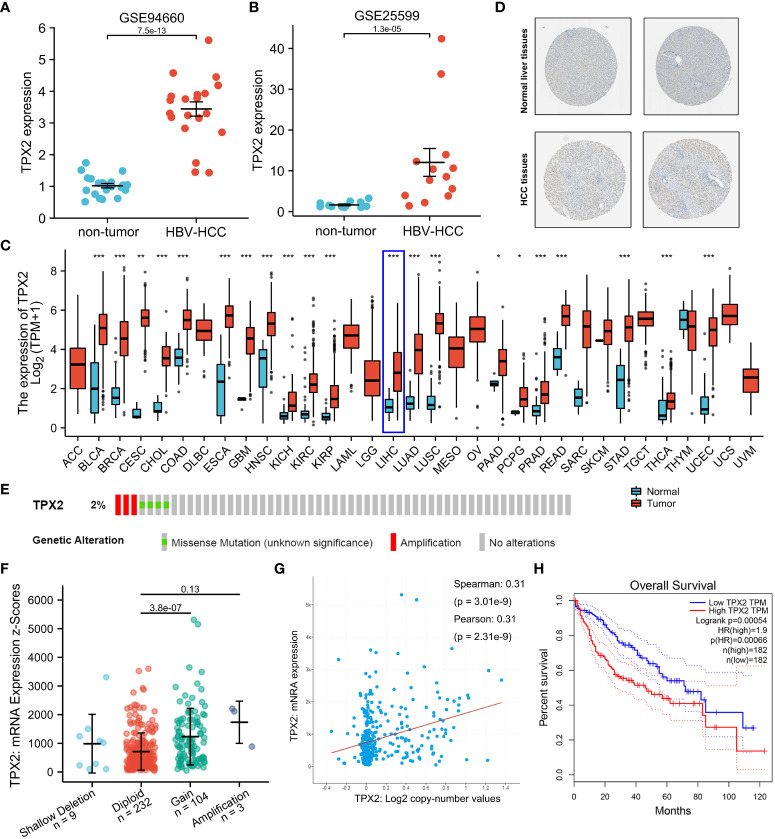
The role of TPX2 in HBV-related HCC. **(A, B)** GEO analysis (GSE25599, GSE94660) of the TPX2 gene expression in HBV-related HCC vs non-tumor groups. **(C)** TCGA analysis of the TPX2 gene expression levels in different tumor samples. **(D)** A validation of TPX2 protein expression in the Human Protein Atlas. **(E)** Heatmap downloaded from the cBioPortal online tool showing TPX2 genetic alternation in HCC samples. **(F, G)** cBioPortal plot analysis of TPX2 mRNA level vs. log2 copy-number value of the TCGA-HCC cohort. **(H)** GEPIA2 analysis of the TCGA-HCC cohort was performed for overall survival between PKIA high expression (50%) group and PKIA low expression (50%) group. *P < 0.05, **P < 0.01, ***P < 0.001.

Previous literature identified TPX2 as a DEG in HBV-infected HCC specimens (Zeng et al., 2020). The raw data (GSE25599 and GSE94660) downloaded from SRA (SRP004768 and SRP099053) was subjected to analysis with limma R package. Firstly, we analyzed the expression level of TPX2 in HBV-related HCC and non-neoplastic liver tissues. As shown in **Figures 2A, B**, based on the GEO datasets, TPX2 shows a higher mRNA level in the HBV-HCC tissues than that in non-tumor groups (p<0.001). In addition, the Human Protein Atlas showed that TPX2 protein expression was significantly higher in HCC tissues than that in normal liver tissues (**Figure 2C**). Since TPX2 is upregulated in HCC tissues, we then screened the expression levels in TCGA pan-cancer RNA sequencing data. The upregulated TPX2 expression patterns in TCGA data are shown in **Figure 2D**. We did obtain a significant difference of TPX2 level for HCC (p<0.001, **Figure 2D**). Next, we investigated the copy numbers of TPX2 in TCGA-HCC dataset using cBioPortal online tool to explore the mechanism of TPX2 upregulation in HCC. Oncoprint module shows that 2% HCC specimens (3/348) have putative copy-number amplification of TPX2 gene (**Figure 2E**). Consistently, low-level gene amplification event (gain copy-number) happens on 30% HCC samples (104/348) compared with TPX2 Diploid group (**Figure 2F**). Notably, TPX2 mRNA expression was significantly correlated with TPX2 copy number values (Spearman Correlation Coefficient = 0.31, p<0.001, **Figure 2G**). The 374 TCGA-HCC cases were divided into low TPX2 and high TPX2 expression groups and the relation of TPX2 expression with the overall survival (OS) was investigated. As shown in **Figure 2H**, HCC cases with higher TPX2 mRNA expression have shown a poor OS (p<0.001). The results indicated that TPX2 was upregulated in HBV-related HCC tissues and associated with poor prognosis of HCC.

### Identification of DElncRNAs, DEmiRNAs and DEmRNAs in TPX2^high^ and TPX2^low^ groups

**Figure 3:**
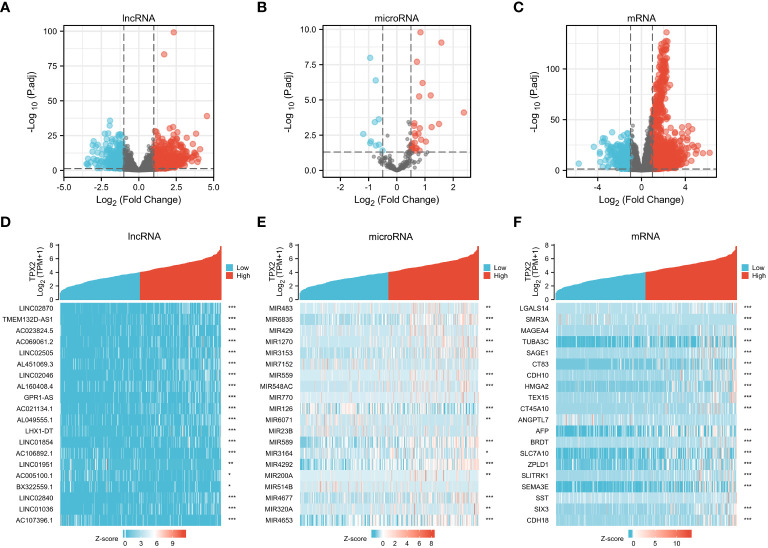
Identification of DElncRNAs, DEmiRNAs and DEmRNAs in TPX2^high^ vs TPX2^low^ groups. **(A–C)** Volcano plot of DElncRNA, DEmiRNAs and DEmRNA in TCGA-TPX2high vs TCGA-TPX2low groups. **(D–F)** Heatmap of the top 20 DElncRNAs, DEmiRNAs and DEmRNAs in TCGA-TPX2^high^ vs TCGA-TPX2^low^ samples. *P < 0.05, **P < 0.01, ***P < 0.001.

A posttranscriptional ceRNA regulatory network contributes to the occurrence and development of several cancers (Qi et al., 2015). Since TPX2 is a worse prognostic biomarker in HBV-related HCC, we aim to investigate the potential downstream network under TPX2 regulation. TCGA-HCC cohort was divided as two group (TPX2^high^ and TPX2^low^) according to the expression level of TPX2. The DElnRNAs between TPX2^low^ and TPX2^high^ samples were screened with the criteria of |LogFC| > 1.5 and adjusted p value < 0.05. We identified 541 DElncRNAs (439 upregulated and 102 down-regulated) between TPX2^low^ and TPX2^high^ groups in the TCGA-HCC dataset (**Figure 3A**). In addition, 37 DEmicroRNAs (27 up-regulated and 10 downregulated) were obtained using a criteria |LogFC| > 0.5 and adjusted p < 0.05 (**Figure 3B**). Applying a cut-off of |LogFC| > 2 and an adjusted p-value < 0.05 for the TPX2^high^-HCC tissues compared with the TPX2^low^-HCC samples, we identified 439 DEmRNAs (381 upregulated and 58 down-regulated) (**Figure 3C**). The heatmap of the top 20 DElncRNAs, DEmicroRNAs and DEmRNAs between TPX2^low^ and TPX2^high^ groups was showed in **Figures 3D–F**.

### Prediction of lncRNA-miRNA-mRNA axis in TPX2-related HCC

**Figure 4:**
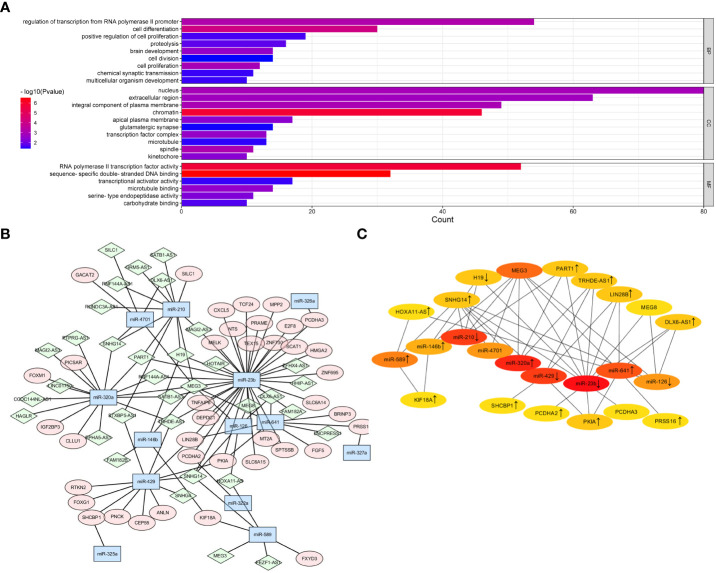
Prediction of lncRNA-miRNA-mRNA network in TPX2-related HCC. **(A)** Construction of ceRNA network for DElncRNAs, DEmiRNAs and DEmRNAs was visualized by Cytoscape. Green diamond indicates lncRNAs. Blue hexagon indicates miRNAs. Pink ellipse indicates mRNAs. **(B)** A CytoHubba module constructed a ceRNA network containing 25 hub genes. **(C)** The GO enrichment plot of DEmRNAs in the TCGA-TPX2^high^ vs TCGA-TPX2^low^ samples.

To investigate the ceRNA network regulated by TPX2 in HBV-HCC, we utilized the DIANA-LncBase v3 (https://diana.e-ce.uth.gr/lncbasev3) online tool to identify miRNAs targeting the 541 DElncRNAs. The results predicted that 33 out of the 541 DElncRNAs interacted with 13 DEmicroRNAs. Next, potential mRNAs targeting the 13 DEmicroRNAs were predicated by using Starbase. Five DEmicroRNAs were predicated to target 46 DEmRNAs. In general, a total of 33 lncRNAs, 13 miRNAs and 46 mRNAs were subjected to Cytoscape software to visualize the lncRNA-miRNA-mRNA network (**Figure 4B**). The top 25 hub genes with nodes degree > 2 were identified and visualized by using CytoHubba module (**Figure 4C**). Finally, the hub regulatory network contained 9 lncRNAs (HOXA11-AS, SNHG14, H19, MEG3, PART1, TRHDE-AS1, LIN28B, MEG8, DLX6-AS1), 9 miRNAs (miR-589, miR-146b, miR-210, miR-4701, miR-320a, miR-429, miR-23b, miR-641, miR-126) and 6 mRNAs (KIF18A, SHCBP1, PCDHA2, PKIA, PCDHA3, PRSS16). Moreover, GO enrichment analysis was performed to investigate the functional annotation of DEGmRNAs and potential cellular function of ceRNA network. **Figure 4A** indicated that the DEmRNAs were significantly enriched in Biological Process terms, including “regulation of transcription from RNA polymerase II promoter”, “cell differentiation”, “cell proliferation”.

### Expression and prognostic value of the hub regulatory network in HCC

**Figure 5:**
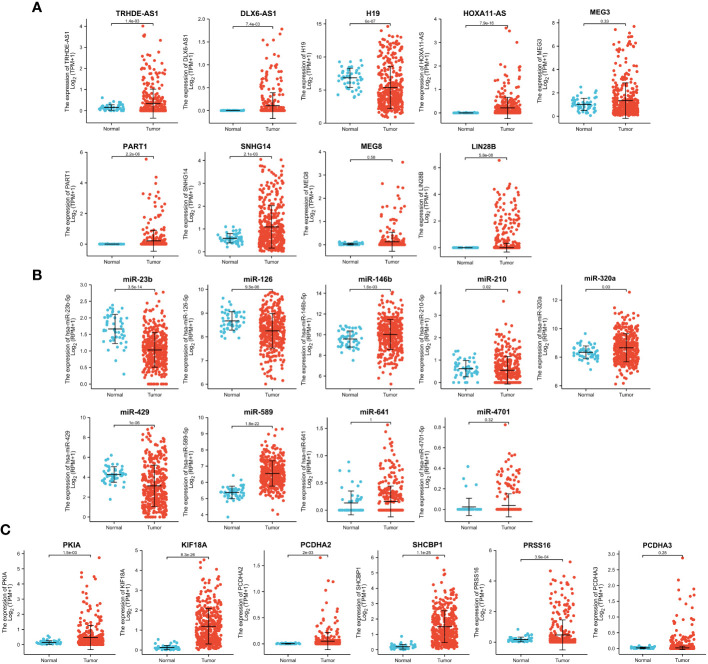
Expression analysis of 25 hub genes in TCGA-HCC. Analysis of TCGA-HCC cohorts showed the expression levels of **(A)** DElncRNAs, **(B)** DEmiRNAs and **(C)** DEmRNAs in normal and tumor groups.

To further screen the candidate ceRNA network, we analyzed the expression of the top 25 hub targets in TPX2^high^-HCC vs TPX2^low^-HCC groups and HCC tumor vs normal groups. As shown in **Figures 5A–C**, the expression levels of six DElncRNAs (TRHDE-AS1, DLX6-AS1, HOXA11-AS, PART1, SNHG14, LIN28B), three DEmiRNAs (miR-146b, miR-320a, miR-589) and five DEmRNAs (KIF18A, SHCBP1, PCDHA2, PKIA, PRSS16) were significantly higher in TCGA-HCC tissues than in normal liver tissues (p < 0.001). In addition, the expression of H19, miR-23b, miR-126, miR-429 and miR-210 was downregulated in HCC tissues as compared to normal tissue (p < 0.01 and p < 0.001). Considering the results in TPX2^high^-HCC vs TPX2^low^-HCC groups, **Table 1** elucidated a consistent finding with the expression analysis of four DElncRNAs (TRHDE-AS1, DLX6-AS1, SNHG14, HOXA11-AS), four DEmiRNAs (miR-23b, miR-320a, miR-589, miR-126) and five DEmRNAs (PKIA, PCDHA2, SHCBP1, PRSS16, KIF18A) in HCC tumor vs normal groups.

**Table 1:** Hub gene expression in TPX2^low^ and TPX2^high^ groups.

gene_name	gene_id	gene_type	log2FC	padj	up/down
MEG3	ENSG00000214548	lncRNA	2.266005503	4.85E-23	up
**TRHDE-AS1**	ENSG00000236333	lncRNA	1.463752992	0.000743	up
**DLX6-AS1**	ENSG00000231764	lncRNA	1.61313044	0.000124	up
H19	ENSG00000130600	lncRNA	1.654348779	5.52E-08	up
PART1	ENSG00000152931	lncRNA	-1.178797661	0.011164	down
**SNHG14**	ENSG00000224078	lncRNA	1.158558875	2.11E-12	up
MEG8	ENSG00000225746	lncRNA	2.576108478	6.94E-13	up
**HOXA11-AS**	ENSG00000240990	lncRNA	1.334528937	3.4E-06	up
**miR-23b**	ENSG00000207563	miRNA	-0.91784113	0.008692	down
**miR-320a**	ENSG00000208037	miRNA	0.807026004	0.039311	up
miR-429	ENSG00000198976	miRNA	1.495517577	0.00051	up
miR-210	ENSG00000199038	miRNA	0.77019038	0.002489	up
miR-641	ENSG00000207631	miRNA	0.680429209	0.02815	up
**miR-589**	ENSG00000207973	miRNA	0.91402209	6.36E-07	up
miR-4701	ENSG00000264201	miRNA	0.542796982	0.033826	up
**miR-126**	ENSG00000199161	miRNA	-0.945521126	1.04E-08	down
miR-146b	ENSG00000202569	miRNA	-0.741363943	0.017269	down
**PKIA**	ENSG00000171033	mRNA	2.03482396	2.58E-20	up
**PCDHA2**	ENSG00000204969	mRNA	2.20983771	3.74E-10	up
**SHCBP1**	ENSG00000171241	mRNA	2.068436367	1.53E-77	up
**PRSS16**	ENSG00000112812	mRNA	2.058319355	5.38E-09	up
**KIF18A**	ENSG00000121621	mRNA	2.231162037	2E-123	up

**Figure 6:**
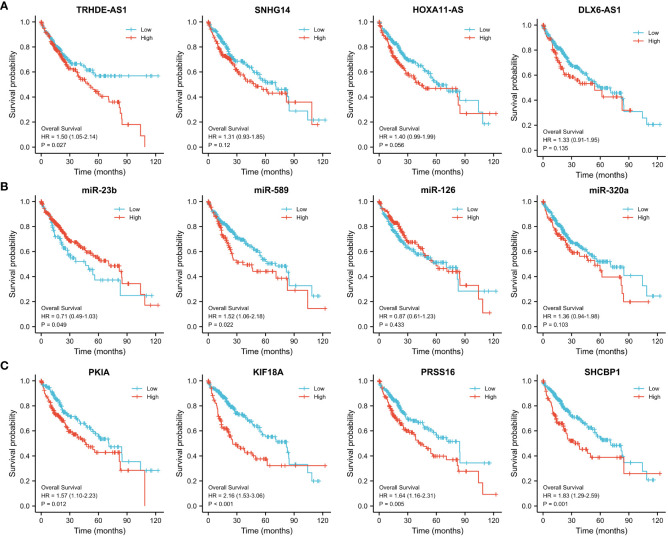
Prognostic value of the hub regulatory network in TCGA-HCC. Overall survival analysis of **(A)** DElncRNAs, **(B)** DEmiRNAs and **(C)** DEmRNAs using Kaplan–Meier survival curves.

Then we investigated the correlation of above lncRNAs/miRNAs/mRNAs expression with the prognosis of patients with HCC, using the dataset of TCGA. As shown in **Figure 6A**, the Kaplan–Meier curves presented a correlation between high expression TRHDE-AS1 and worse overall survival (p < 0.05). However, low expression of miR-23b and miR-589 was associated with poor prognosis (p < 0.05, **Figure 6B**). Additionally, the high expression of PKIA, SHCBP1, PRSS16 and KIF18A worsened the overall survival in HCC (p < 0.05, p < 0.001, **Figure 6C**). In brief, one DElncRNA (TRHDE-AS1), two DEmiRNAs (miR-23b and miR-589), and four DEmRNAs (PKIA, SHCBP1, PRSS16 and KIF18A) were found to be prognostic biomarkers in HCC.

### Validation of TRHDE-AS1//PKIA network in HCC

**Figure 7:**
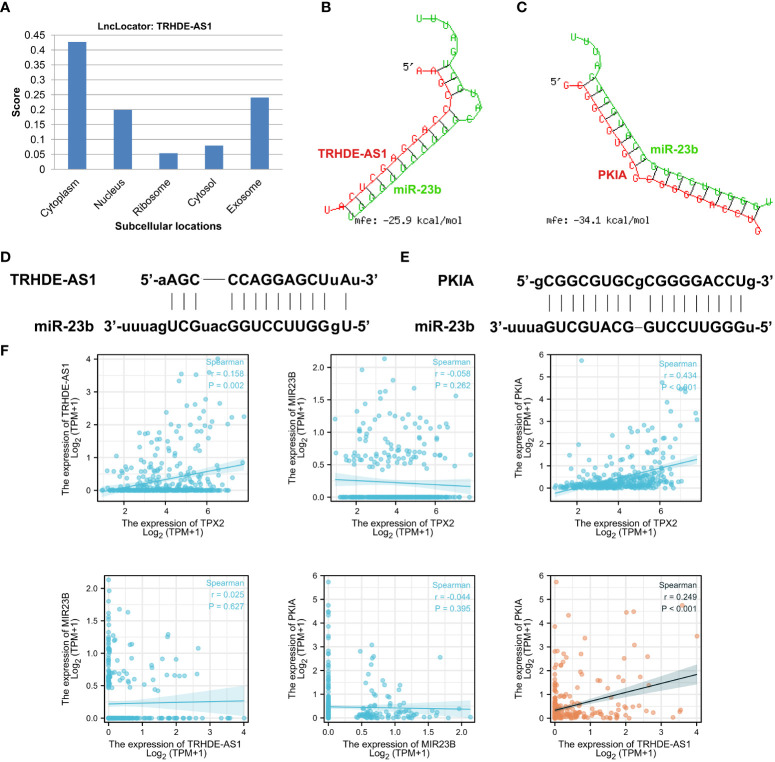
Validation of TRHDE-AS1/miR-23b/PKIA network in HCC. **(A)** Subcellular locations analysis of lncRNA TRHDE-AS1 using LncLocator. **(B, C)** RNAhybrid was utilized to predict the binding between TRHDE-AS1/PKIA and miR-23b. **(D, E)** Schematic diagram showing the predicted binding sites between miR-23b and TRHDE-AS1/PKIA. **(F)** Correlation analysis between RNAs and RNAs or between TPX2 and RNA.

To determine a specific ceRNA network, the candidate lncRNA TRHDE-AS1 was subjected to a further analysis. Firstly, a lncRNA subcellular localization predictor (LncLocator online tool) was utilized to predict the location of TRHDE-AS1 in human cells. As shown in **Figure 7A**, lncRNA TRHDE-AS1 accumulates in the cytoplasm and may act as a ceRNA to sponge miRNA. Next, RNAhybrid was used to predict the binding sites between TRHDE-AS1 and miRNA. A putative miR-23b binding site was identified in the regions of TRHDE-AS1 with an minimum free energy (mfe) -25.9 kcal/mol (**Figure 7B**). However, there is not a binding site between TRHDE-AS1 and miR-589. RNAhybrid algorithm predicted the interaction between miR-23b and PKIA, not other three prognostic DEmRNAs (**Figure 7C**). Schematic diagram showed the predicted binding sites between miR-23b and TRHDE-AS1/PKIA (**Figures 7D, E**). Correlation analysis showed that TRHDE-AS1 expression is positively correlated with PKIA in TCGA-HCC cohort (r = 0.249, p < 0.001). However, there was not significance between miR-23b expression and PKIA levels. These results indicated that TRHDE-AS1/PKIA axis is a potential ceRNA network in HCC.

### Validation of PKIA methylation in HCC

**Figure 8:**
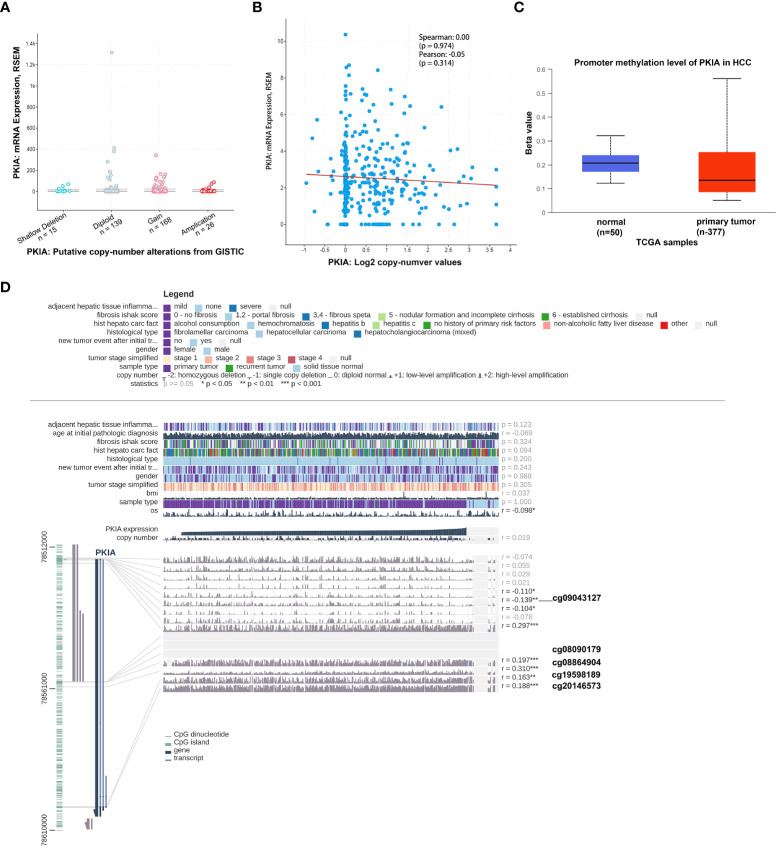
Validation of PKIA methylation in HCC. **(A, B)** cBioPortal plot analysis of PKIA mRNA level vs. log2 copy-number value of the TCGA-HCC cohort. **(C)** UALCAN online data analysis platform analyzing the methylation on PKIA promoter. **(D)** MEXPRESS visualization tool showing the potential PKIA DNA methylation sites.

Next, we further explored the potential mechanism regulating PKIA expression in HCC. As shown in **Figure 8A**, the genetic alteration status of PKIA did not appear in HCC samples of the TCGA cohorts. Moreover, we observed that there is not a potential association between copy-number of PKIA and expression level of PKIA mRNA (**Figure 8B**). Thus we hypothesized that the abnormal expression of PKIA might be related to DNA methylation in HCC. UALCAN online data analysis platform (http://ualcan.path.uab.edu/) evaluated epigenetic regulation of PKIA expression by promoter methylation. The results showed a methylation difference between primary tumor and normal tissues (**Figure 8C**). In addition, the data analysis in PKIA^high^ and PKIA^low^ groups showed that the levels of methylation-related genes (DNMT1, DNMT3A, DNMT3B) were downregulated in PKIA^high^-TCGA samples, compared with PKIA^low^-TCGA group (p < 0.001, **Table 2**). Furthermore, the MEXPRESS visualization tool (https://mexpress.be/) was utilized to investigate the potential PKIA DNA methylation. A negative correlation of PKIA level and DNA methylation at the promoter region (probe ID: cg09043127) was shown in **Figure 8D** (p < 0.01). These results suggested that the DNA methylation of PKIA is responsible for PKIA upregulation, which provided a potential mechanism to understand the ceRNA network regulation in HCC.

**Table 2:** Expression level of methylation markers in PKIA^low^ and PKIA^high^ groups.

gene_name	gene_id	log2FC	padj	significance
DNMT1	ENSG00000130816	-0.572	7.07E-11	***
DNMT3A	ENSG00000119772	-0.417	3.6E-06	***
DNMT3B	ENSG00000088305	-0.737	1.13E-10	***

***p < 0.001.

### Analysis of relationship between PKIA and immune infiltration in HCC

**Figure 9:**
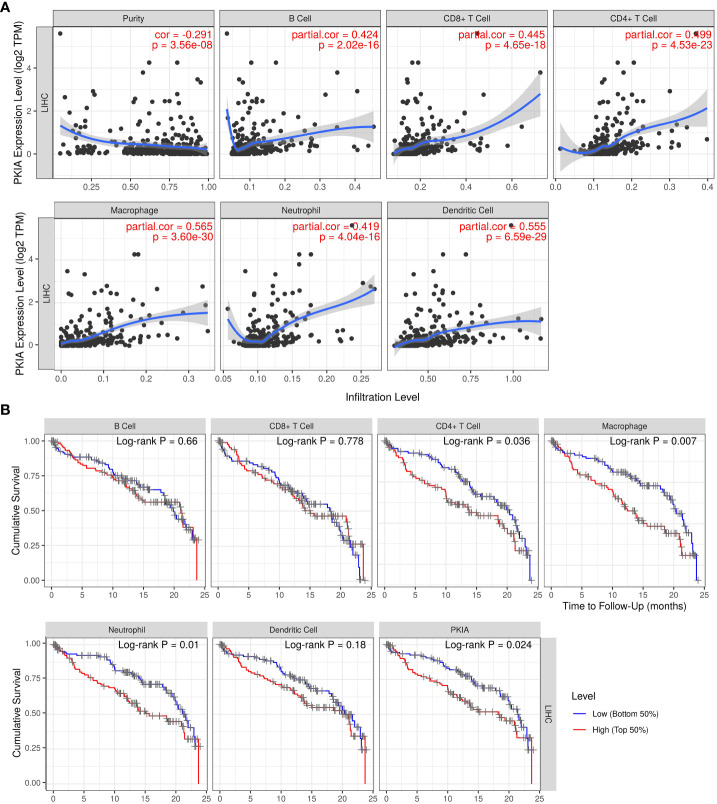
Analysis of relationship between PKIA and immune infiltration in HCC. **(A)** Correlation analysis between PKIA expression and infiltrating levels of B cells, CD8+ T cells, CD4+ T cells, macrophages, neutrophils and dendritic cells in HCC. **(B)** Survival analysis between PKIA expression and immune cells in HCC.

As is well-known that immune cell infiltrating is an essential factor for cancer patient’ survival. The above analysis indicated that TRHDE-AS1/PKIA axis can be important prognostic role in HCC. We then explored the effect of PKIA expression on immune infiltration in HCC. The results from TIMER showed that PKIA level had a obvious negative correlation with tumor purity in HCC (**Figure 9A**). After adjusting with tumor purity, PKIA expression levels were significantly correlated with the infiltration levels of B cells (cor = 0.424, p = 2.02e-16), CD8+ T cells (cor = 0.445, p = 4.65e-18), CD4+ T cells (cor = 0.499, p = 4.53e-23), macrophages (cor = 0.565, p = 3.60e-30), neutrophils (cor = 0.419, p = 4.05e-16), and dendritic cells (cor = 0.555, p = 6.59e-29). In addition, PKIA gene expression was significantly correlated with the infiltration levels of 49 immune cell markers in HCC (**Table 3**). To explore the clinical relevance of several tumor immune subsets in a Cox proportional hazard model, TIMER algorithm was utilized for estimating the abundances of immune infiltrates and survival in HCC. As shown in **Figure 9B**, the high abundances of CD4+ T cell (p < 0.05), macrophage (p < 0.01), neutrophil (p < 0.01). The results indicated that immune infiltrates in HCC was associated with worse prognosis.

**Table 3:** Correlation analysis between PKIA and immune infiltration biomarkers in HCC.

Description	Gene markers	LIHC		
		correlation	p value	significance
CD8+ T cell	CD8A	0.488	1.29E-23	***
	CD8B	0.448	9.36E-20	***
T cell	CD3D	0.570	2.72E-33	***
	CD2	0.557	1.21E-31	***
B cell	CD19	0.470	8.31E-22	***
	CD79A	0.471	6.82E-22	***
Monocyte	CD86	0.603	4.62E-38	***
	CSF1R	0.508	1.01E-25	***
	CD86	0.603	4.62E-38	***
TAM	CCL2	0.439	6.26E-19	***
	CD68	0.451	5.16E-20	***
	IL10	0.467	1.59E-21	***
M1 macrophage	NOS2	0.061	0.242514208	ns
	IRF5	0.312	8.02E-10	***
	PTGS2	0.440	1.50E-36	***
M2 macrophage	VSIG4	0.410	1.76E-16	***
	MS4A4A	0.405	4.75E-16	***
Neutrophils	CEACAM8	0.127	0.014402811	*
	ITGAM	0.455	2.54E-20	***
	CCR7	0.438	8.32E-19	***
Natural killer cell	KIR2DL1	0.046	0.373942946	ns
	KIR2DL3	0.231	6.87E-06	***
	KIR2DL4	0.269	1.40E-07	***
	KIR3DL1	0.076	0.143874889	ns
	KIR3DL2	0.241	2.56E-06	***
	KIR3DL3	0.096	0.064199312	ns
	KIR2DS4	0.166	0.001346004	**
Dendritic cell	HLA-DPB1	0.487	1.74E-23	***
	HLA-DQB1	0.415	7.07E-17	***
	HLA-DRA	0.445	1.81E-19	***
	HLA-DPA1	0.469	1.03E-21	***
	CD1C	0.421	2.44E-17	***
	NRP1	0.382	2.63E-14	***
	ITGAX	0.561	4.18E-32	***
Th1	TBX21	0.386	1.24E-14	***
	STAT4	0.371	1.52E-13	***
	STAT1	0.430	4.32E-18	***
	IFNG	0.400	1.11E-15	***
	TNF	0.514	2.23E-26	***
Th2	GATA3	0.583	3.33E-35	***
	STAT6	0.154	0.003022523	**
	STAT5A	0.484	3.23E-23	***
	IL13	0.099	0.056678881	ns
Tfh	BCL6	0.159	0.002096074	**
	IL21	0.170	0.001009247	**
Th17	STAT3	0.265	2.34E-07	***
	IL17A	0.089	0.088102091	ns
Treg	FOXP3	0.254	7.17E-07	***
	CCR8	0.526	9.66E-28	***
	STAT5B	0.166	5.61E-06	***
T cell exhaustion	PDCD1	0.553	4.13E-31	***
	CTLA4	0.570	2.17E-33	***
	LAG3	0.359	1.07E-12	***
	HAVCR2	0.613	1.35E-39	***
	GZMB	0.315	5.73E-10	***

ns, not significance, *P < 0.05, **P < 0.01, ***P < 0.001.

## Discussion

Emerging publications have reported that TPX2 overexpression is closely related to the development of various malignant tumors, including cervical cancer, esophageal squamous cell carcinoma and pancreatic cancer (Chang et al., 2012; Hsu et al., 2014; Gomes-Filho et al., 2020). In this study, our GEO and TCGA analysis results also confirmed the overexpression of TPX2 in HBV-related HCC tissues and an amplification of TPX2 DNA copy-number alterations (CNA) in HCC, suggesting that CNA might contribute to upregulation of TPX2 among HCC samples. Interestingly, the clinical RNA-seq-based evidence supports the role of TPX2 expression in prognosis of HCC. However, the regulatory mechanism which TPX2 plays a role in tumorigenesis of HBV-HCC remains to be answered. We comprehensively examined DElncRNAs, DEmiRNAs and DEmRNAs based on the expression level of TPX2 in HBV-HCC to explore a potential ceRNA network.

The function of lncRNAs in the occurrence and development of HCC is closely related to its structural characteristics and its role in the biological process (Zhang et al., 2020). LncRNAs participate in majority processes of gene regulation, including genome transcription, mRNA splicing and RNA attenuation (Peng et al., 2017; Gil and Ulitsky, 2020). In the study, TCGA-HCC database was utilized to identify DElncRNA, DEmiRNAs and DEmRNAs between TPX2^high^ vs TPX2^low^ groups, so as to screen a TPX2-related ceRNA network. Based on the RNA-RNA interaction, the DEmiRNAs obtained from DIANA-LncBase v3 and Starbase tools are intersected to obtain the granularity common miRNAs. We constructed a primary lncRNA-miRNA-mRNA network which contains a total of 33 lncRNAs, 13 miRNAs and 46 mRNAs. Considering the results from Cytohubba hub genes, common DEGs in tumor vs normal groups and DEGs in TPX2^high^ vs TPX2^low^ groups was subjected to prognosis analysis in HCC. The common DEGs included four DElncRNAs (TRHDE-AS1, DLX6-AS1, SNHG14, HOXA11-AS), four DEmiRNAs (miR-23b, miR-320a, miR-589, miR-126) and five DEmRNAs (PKIA, PCDHA2, SHCBP1, PRSS16, KIF18A. Several reports have indicated the roles of these DElncRNAs in tumor pathogenesis. LncRNA DLX6-AS1 acting as a miR-513c sponge was found to promote the tumor malignancy progression of HCC through modulating Cul4A/ANXA10 (Liu et al., 2021). LncRNA SNHG14 regulated PTEN through activating PABPC1, which can aggravate cell proliferation and angiogenesis of HCC cells (Zhang et al., 2020). LncRNA HOXA11-AS promoted the HCC cell proliferation and EMT process with a mechanism in which HOXA11-AS may act as a ceRNA by directly sponging miR-506-3p8 to regulate the Slug expression (Liu et al., 2020). In this comprehensive research, we first presented evidence of TRHDE-AS1 upregulation in HCC and the potential correlation between TRHDE-AS1 expression and prognosis across TCGA-HCC cohort and TPX2-related HCC.

LncRNAs can act as miRNA sponges, recognizing cytosolic miRNAs and thereby regulating the expression of miRNA targeting gene (Paraskevopoulou and Hatzigeorgiou, 2016). In addition to the function as ceRNAs, lncRNAs also can compete with miRNAs for binding to target mRNAs and control a variety of cellular processes, including cell proliferation, differentiation, immune responses, angiogenesis and inflammation (Schmitz et al., 2016). In this study, we confirmed the abnormal expression levels of miR-23b and miR-589 in HCC, as well as their prognostic patterns. miR-589, likely expressed in HCC tissues, played a functional important role in prognostic signature and was recently proved to involve in HCC cell growth (Xu et al., 2018). Previous report suggested that miR-23b was down-regulated obviously in clinical HCC tissues and its exogenous upregulation inhibited EMT process of HCC cells (Cao et al., 2017; He et al., 2018). In this research, we show a schematic diagram indicating the bind sites between TRHDE-AS1 and miR-23b. However, no significant correlation was found between TRHDE-AS1 expression and miR-23b level in TCGA-HCC database.

Moreover, the interaction between miR-23b and downstream target mRNA was also found by using Starbase online tool. The mRNA 3’UTR- of cAMP-dependent protein kinase inhibitor alpha (PKIA) is predicted to bind with miR-23b. Although osteosarcoma patients with low PKIA expression was reported to have poor prognosis (Lin et al., 2022), our TCGA-based expression and survival analysis results indicated a correlation between PKIA high expression and worse prognosis for HCC patients. A report has suggested the role of over-expressed PKIA in the tumor growth of prostate cancer (Hoy et al., 2020) and proposed gene amplifications of PKIA across in prostate and lung cancers (Hoy et al., 2020). However, our analysis showed a minor alterations in HCC and no correlation between PKIA copy-number and PKIA mRNA expression. Interestingly, for HCC patients of TCGA, we observed a reduced DNA methylation status in primary HCC samples, which give a evidence for the upregulation of PKIA in HCC. Moreover, we found that TRHDE-AS1 expression level was positively correlated with PKIA level in HCC.

Next, we further interpreted the function of TRHDE-AS1/PKIA network in TPX2-related HCC and elucidated the potential mechanism of TRHDE-AS1/PKIA network in HCC patient prognosis. Meanwhile, HBV replication activates the chronic inflammation, immune response mechanisms and related inflammatory pathways, leading to the recruitment of NK cells, NKT cells, and cytotoxic T cells (Tang et al., 2018). Hepatocytes infiltrate into the microenvironment of related inflammatory cytokines, trigger liver cell damage and indirectly induce HCC (Lim et al., 2019; Jia et al., 2020). We utilized TIMER online tool which contains multiple immune deconvolution methods to analyze the correlation between PKIA expression and the immune infiltration levels. TAMs can promote tumor malignancy by producing cathepsins and facilitate cancer cell migration and invasion (Pathria et al., 2019). In this research, the expression levels of tumor-associated macrophages (TAMs)-related markers (CCL2, CD68 and IL10) were a positive correlated PKIA expression, indicating an ontogenetic role of PKIA in HCC.

Taken together, this study constructed a ceRNA network containing TRHDE-AS1 and PKIA, which related to TPX2 regulation in the HBV-related HCC. In addition, TRHDE-AS1/PKIA network also participated in prognosis of HCC patients. However, more research on the function of TRHDE-AS1 in HBV-infected HCC cells needs to be undertaken before the association between TRHDE-AS1 and PKIA is more clearly understood. Moreover, further experimental verification will be carried out to confirm the binding sites between TRHDE-AS1/PKIA and miR-23b in HCC.

## Data availability statement

The datasets presented in this study can be found in online repositories. The names of the repository/repositories and accession number(s) can be found in the article/supplementary material.

## Author contributions

Conceptualization, GL, ZQW, DC, and JM. Resources, GL, ZQW, JY, ZM, BS, and JM. Data curation, GL, TY, XZ, ZZ, ZQW, and JM. Software, GL, ZQW, DC, YL, PC, and YD. Formal analysis, GL, ZQW, DC, and JM. Supervision, JY, ZM, BS, YL, PC, and YD. Funding acquisition, JM. Validation, GL and JM. Visualization, GL, ZMW, DC, JY, and ZM. Methodology, TY, XZ, ZZ, ZMW, and JM. Writing—original draft, GL. Writing—review and editing, JM. All authors read and approved the final manuscript. All authors contributed to the article and approved the submitted version.

## Funding

This study was funded by Shanxi Scholarship Council of China (Grant No: 2021-165), Shanxi Province Science Foundation for Distinguished Young Scholar (Grant No: 201901D211547), science and research fund of Shanxi Health Commission(Grant No: 2019059, 2022042, 2022043), Shanxi Province”136 Revitalization Medical Project Construction Funds”, National Natural Science Foundation of China for Young Scholars (Grant No: 81201810), the doctor project of Shanxi Cancer Hospital, China (2017A06), Natural Science Foundation of Guangdong Province, China (2015A030313057).

## Conflict of interest

The authors declare that the research was conducted in the absence of any commercial or financial relationships that could be construed as a potential conflict of interest.

## Publisher’s note

All claims expressed in this article are solely those of the authors and do not necessarily represent those of their affiliated organizations, or those of the publisher, the editors and the reviewers. Any product that may be evaluated in this article, or claim that may be made by its manufacturer, is not guaranteed or endorsed by the publisher.
